# Maintenance versus replacement of medical equipment: a cost-minimization analysis among district hospitals in Nepal

**DOI:** 10.1186/s12913-022-08392-6

**Published:** 2022-08-12

**Authors:** Michael Hillebrecht, Constantin Schmidt, Bhim Prasad Saptoka, Josef Riha, Matthias Nachtnebel, Till Bärnighausen

**Affiliations:** 1 Heidelberg Institute of Global Health (HIGH), Medical Faculty and University Hospital, Heidelberg University, Heidelberg, Germany; 2 South-Asia Institute, Heidelberg University, Heidelberg, Germany; 3 Deutsche Gesellschaft für Internationale Zusammenarbeit (GIZ) GmbH, Eschborn, Germany; 4 Health Coordination Division, Ministry of Health and Population, Kathmandu, Nepal; 5 Division of Infectious Diseases and Tropical Medicine, Center for International Health, University Hospital Munich, Ludwig-Maximilians-University, Munich, Germany; 6 Management4health, Frankfurt, Germany; 7 Health and Social Protection Asia, KfW Development Bank, Frankfurt am Main, Germany; 8 Africa Health Research Institute (AHRI), Somkhele, KwaZulu-Natal South Africa; 9 MRC/Wits Rural Public Health and Health Transitions Research Unit (Agincourt), School of Public Health, Faculty of Health Sciences, University of the Witwatersrand, Johannesburg, South Africa; 10 Center for Population and Development Studies, Harvard University, Cambridge, MA USA

**Keywords:** Nepal, Maintenance, Health management, Health technology, Asset management, Costing study

## Abstract

**Background:**

About half of all medical devices in low- and lower-middle-income countries are currently non-operational because equipment maintenance is lacking. Thus, choosing a cost-efficient equipment maintenance approach has the potential to increase both the quantity and quality of important health services. Between 2010 and 2014 Nepal’s Ministry of Health chose two of its development regions to pilot the contracting-out of maintenance services to the private sector. We develop a cost model and employ different data to calculate the cost of this contracted-out scheme. The latter we compare with two additional common approaches to maintenance: in-house maintenance and no maintenance.

**Methods:**

We use invoiced pilot program costs, device depreciation estimates from the literature, and hospital case numbers from Nepal’s Health Management Information System. We estimate net-present values for a three-year horizon, incorporating both fixed and operational cost. Operational costs include downtime cost measured as lost revenues due to non-working equipment.

**Results:**

The contracted-out maintenance scheme shows a strong relative cost performance. Its cost after 3 years amount to 4,501,574 International Dollars Purchasing Power Parity (I$ PPP), only 90% of the cost with no maintenance. The contracted-out scheme incurs 670,288 I$ PPP and 3,765,360 I$ PPP in fixed cost and operational cost, respectively. The cost for replacing broken devices is 1,920,467 I$ PPP lower with maintenance. In addition, after 3 years total cost of contracted-out maintenance is 489,333 I$ PPP (11%) below total cost of decentralized in-house maintenance. After 10 years, contracted-out maintenance saves 2.5 million I$ PPP (18%) compared to no maintenance.

**Conclusions:**

We find that contracted-out maintenance provides cost-efficient medical equipment maintenance in a lower-middle income context. Our findings contrast with studies from high- and upper-middle-income countries, which reflect contexts with more in-house engineering expertise than in our study area. Since the per hospital fixed cost decrease with scheme size, our results lend support to an expansion of contracted-out maintenance to the remaining three development regions in Nepal.

**Supplementary Information:**

The online version contains supplementary material available at 10.1186/s12913-022-08392-6.

## Background

Modern medicine is unthinkable without medical devices, such as X-rays, ultrasound machines or microscopes. They facilitate the prevention, diagnosis, and treatment of illnesses and as such directly affect human lives. At the same time, the acquisition and ownership of medical devices is expensive – including considerable cost of maintenance – and competes with other investments targeted towards quality health care provision. Choosing the right approach to manage and maintain medical equipment thus might come with substantial efficiency and performance gains for a country’s health care system [1]. However, comprehensive medical equipment maintenance policies are rare in the Global South [2, 3]. Challenges include the absence of funding for maintenance schemes among the international donor community [4], miss-management, for instance, through misallocation of spare parts or non-aligned purchases of medical devices [3, 4], as well as the lack of qualified technical staff [4–6].

In addition, scientific evidence on the performance and effects of medical equipment maintenance schemes in low and lower-middle income countries is scarce. A set of studies investigates the current state of equipment maintenance by building up inventories and conducting expert interviews to assess equipment operability and qualitative maintenance management recommendations [2, 5, 7–9]. Two studies assess the performance of medical equipment schemes in an empirical fashion [6, 10]. Yet, to the best of our knowledge, the literature provides no answers to the question whether medical equipment maintenance delivers long-term cost savings and quality of care improvements from a health system perspective.

Thus, our paper focuses on the cost dimension and aims to provide novel evidence on several research gaps. First, it provides the first detailed cost analysis of a contracted-out medical equipment maintenance scheme covering multiple hospitals in a lower-middle income country context. Second, it includes the first – albeit hypothetical - comparative cost efficiency assessment of the three most common maintenance modes: contracted-out, in-house, and no maintenance. Last, this study makes a methodological contribution by including downtime cost into the equation. We use a simple costing model with fixed, operational, and downtime cost to estimate the net present value under these three different maintenance scenarios.

Nepal, the context of this study, provides a showcase for the successful stepwise adaption of a maintenance management system, covering medical equipment of medium complexity (such as X-ray and ultrasound). Based on an initial equipment inventory and external donor support, the government delegated responsibility for equipment maintenance services, as well as trainings for in-house staff, to a private contractor. Before the adaptation of the maintenance system, non-operational devices were simply replaced with new equipment, which is still the reality in many low-income countries.

## Methods

### Pilot intervention

In the fiscal year 2009/10, Nepal’s Ministry of Health operated 65 primary care hospitals (district hospitals) and 27 higher level hospitals (zonal, regional, and central hospitals) [11]. The public health system was financed through the central state government budget, contributions at point of service – free services were provided to poor and vulnerable populations – and international donors. Our study analyzes the pilot program of Nepal’s national contracted-out maintenance scheme for medical equipment of medium complexity. The scheme was run by the Ministry of Health with financial support from the German KfW Development Bank and technical assistance from management4health, an international consultancy. In addition, researchers from Heidelberg University’s Institute for Global Health conducted an independent ex-post review of how the program was implemented. We draw on data generated over the program’s pilot between 2010 and 2014 in Nepal’s Far-Western and Mid-Western region. As no inventory of medical devices in public hospitals existed before the pilot, an initial rapid inventory assessment was conducted. Based on this assessment, the government’s Physical Assets Management unit commissioned both preventive and corrective maintenance services for all medical devices of medium complexity located in public regional, sub-regional, zonal, and district hospitals. Our study focuses on the 19 district hospitals which cover an average population of 291,000 individuals, each.

In addition, the contractor’s service package covered maintenance of cold chain equipment in district health offices and equipment-related training to hospital staff. Inventory management and procurement of new devices remained under the responsibility of the Ministry of Health. In total, the contractor engaged 14 employees on this job (managing director, four biomedical engineers, six biomedical engineering technicians and three logistics managers), of which the majority (except the managing director, the chief engineer and one logistics manager) was assigned to one of the two regional maintenance workshops, in Nepalgunj (Mid-West) and Danghadhi (Far-West). Biomedical engineering technicians were trained at BMET Training Centre in Kathmandu and provided the first line of service, by moving between workshops and hospital facilities as required. Biomedical engineers provided backstopping. Job assignments were decided by the respective workshop manager as per maintenance schedule, job urgency and expertise required.

During the mobilization phase, the contractor visited all hospitals for a thorough inspection and the creation of a final list of devices. Maintenance activities over the contract period of three years included six semi-annual maintenance visits for non-critical devices. Critical devices (such as X-ray, operating lights, electrosurgical units, sterilizing units in higher-than-district-level hospitals) received three preventive maintenance visits per year. In addition, the contract included the provision of corrective maintenance services, with different support response rates being offered for critical and non-critical devices. To monitor maintenance activities and payments, the Physical Asset Management unit centrally established a digital maintenance management information system (MMIS) prior to the mobilization phase. It records all maintenance and repair activities through the continuous collection of so-called “jobcards” and thereby provides an up-to-date device inventory.

### Data

Nepal’s Health Management Information System (HMIS) collects a wide range of health provider information on a regular basis. It reports monthly data on patient numbers, services delivered, and performance indicators for all public health facilities. The data management unit of the Ministry of Health gathers and publishes HMIS data to facilitate administrative procedures and to inform policy decisions. Our data contain case numbers of six equipment-based health services, which we extract for the two reporting rounds 2008/09 and 2014/15 (Table 1). To put HMIS headcounts into perspective, we relate district hospital case numbers to the population of respective catchment areas. For medical equipment downtime cost calculation we obtained remuneration rates for equipment-based health services from Nepal’s national Social Health Security Program [12].

**Table 1:** Summary statistics of device types included in the analysis

			1	2		3	4	5	6
			Devices total	Devices per HF		Total value (I$ [PPP], 2016)	Number of Services^a^	Corrective Maintenance°	Device Lifetime
				Mean	SD				
**1**	**Laboratory equipment**								
	a	Analyzer	11	0.6	1.13	292,169	55.64	5	7.5
	b	Centrifuge	53	2.9	1.08	125,785	(Analyzed in laboratory^b^)	41	9.0
	c	Microscope	41	2.3	1.07	311,377		19	10.0
**2**	**Monitoring**								
	a	ECG	21	1.2	0.79	247,203	0.19	14	8.0
**3**	**Ultrasound imaging**		50	2.8	1.07	2,230,901	5.39	9	10.0
**4**	**X-ray imaging**		47	2.6	1.54	6,065,638	9.06	73	8.0
	**Total**		223	12.4		9,273,072	70.28	161	

*Notes*: Numbers for hospitals included into our costing study. Abbreviations: ecg, electrocardiogram. ^a^Per 1,000 inhabitants, per year. Average values over 2010/11 till 2013/14 Health Management Information System rounds. ^b^Number of cases examined in the laboratory using one or more of analyzer, centrifuge, or microscope. °Total number of corrective maintenance visits over six rounds. Data sources: Devices total and Devices per HF, Health Management Information System; Total value, Consultant; Number of Services and Corrective Maintenance, HMIS; Device Lifetime, engineering literature [12, 13]

From within Nepal’s pilot program, we draw on two sources of information. First, we use records from preventive and corrective maintenance activities and the number of devices from the program’s MMIS. As the inclusion of downtime cost requires the combination of MMIS and HMIS data, our merged dataset for analysis covers six medical devices out of the total 15 covered in the MMIS. For considerations of space and easier comparability this study focuses on district hospitals, only. Second, we include invoiced maintenance services and other cost accounts from the pilot project’s implementation phase.

Table 1 shows descriptive statistics from our set of 19 district hospitals, for our selection of devices. In total we include 223 devices, or about 13 devices per hospital. Due to differences in hospital size, the number of devices varies greatly across hospitals. Similarly, there are large differences in equipment values. In total, all 53 centrifuges cost about 47,000 International Dollar Purchasing Power Parity (I$ PPP) while the sum of all 47 X-rays amounts to about 2.25 million I$ PPP. Laboratory equipment use is frequent with around 56 services per 1000 inhabitants per year. ECG, in contrast only provides 0.2 services per 1000 inhabitants. On average, each device required six corrective maintenance visits per year.

### Cost equation

Taking a health system planner perspective, our cost evaluation is a comparative assessment of three maintenance scenarios (contracted-out maintenance, in-house maintenance, and no maintenance) including cost at the hospital and central management level. We employ a costing model with fixed and operating costs, as set out in eq. (1). Fixed costs are composed of establishing the central administrative unit and either building in-house workshops or setting up the contracted-out scheme. Operating costs accrue for central maintenance system management, device replacements and maintenance operations. Our model also accounts for downtime as an additional operational cost component, to control for lost hospital revenues due to a lower share of operational devices. Our outcome indicator is the net present value of total cost during the length of the pilot (three years), for which we assume a discount rate (*r*) of 5%. Prices were adjusted for inflation and local currencies, and converted into I$ PPP using the World Bank’s World Development Indicators [13]. Please see in Table A9 in the appendix for detailed information.

1 $$C={C}^{Fixed}+{C}^{Operating}={C}^{Fixed}+\sum \limits_{t=1}^T\frac{{C_t}^{Management}+{C_t}^{Replacement}+{C_t}^{Maintenance}+{C_t}^{Downtime}}{{\left(1+r\right)}_t}$$

### Cost components

Replacement cost is the average expected investment in new devices based on purchasing value and number of devices. For instance, if a device has a lifetime of 10 years, the hospital should expect average yearly replacement costs of one tenth the total purchasing value of its current stock of this device. A cross-country delphi study provides lifespan intervals for four different equipment categories, distinguishing between good and bad quality devices, as well as between good and bad maintenance [14]. Based on these intervals we have calculated an average lifetime reduction rate incurred by applying bad (or no) maintenance instead of good maintenance, amounting to 39%.

To the best of our knowledge, downtime cost has not been included into costing studies of medical equipment maintenance, yet, even if they might constitute a substantial share of overall equipment-related cost [15]. At the hospital level, the cost of equipment downtime are missed profits due to fewer equipment-related services offered and reimbursed. From a health system perspective, additional costs may accrue as non-functional equipment will result in inadequate diagnosis and therapy, thereby hampering quality of care and potentially harming patients [16]. A program’s ability to reduce equipment downtime thus may, by itself, provide a useful hospital performance benchmark measure for comparison. We therefore deliberately present and discuss downtime cost differences across maintenance modes. However, we are aware that with our hospital-focused approach of downtime calculation we provide rather lower bound estimates for the true economic cost related to an increase in equipment downtime.

We define equipment downtime as the number of days a device is unavailable for services due to technical defects. We calculate downtime from the time passed between the request for a corrective maintenance task (as recorded in the MMIS) and task completion. Since it is very unlikely that a request is filed immediately after the device breaks down, we consider 15% of the time passed between a corrective maintenance job request and the equipment’s last inspection, whether it was scheduled or not, as additional downtime. We used downtime from the year before the maintenance pilot was started as a benchmark for downtime under no maintenance. We provide a robustness check for this assumption in Table A5 in the Appendix. From HMIS reports we then extract the number of services provided by each device type in an average-sized district hospital per day. Next, we assign a price tag to each service by employing health services remuneration rates from Nepal’s National Social Health Security Program. These numbers reflect rates charged by public hospitals and, depending on the service, range from 3.0 to 13.5 I$ PPP per service. As such, they provide a lower bound of the health services’ true market value. By multiplying the number of days a device is unavailable with the number of services per day and renumeration rates, we estimate the annual downtime cost for each device. Table A1, in the Appendix, provides a definition of each cost type and the respective data source.

### Cost scenarios

The benchmark scenario for our study is contracted-out maintenance, which was piloted in our study area. Accordingly, cost estimates mainly come from invoiced program costs and additional figures from the literature (column 2 in Table 2). Due to mentioned data constraints, we had to narrow down the analysis to six device types. Therefore, we reduce IT, transport, and coordination components of management cost accordingly. We keep other management cost the same, like the number of rooms and the initial inventory. We split the contracted-out cost of the mobilization phase and the pilot by number of devices. Table A2 in the Appendix provides a detailed description of the assumptions we made.

**Table 2:** Maintenance scenarios and corresponding program cost

Cost scenario:	A	B		C
	No maintenance	Contracted-out maintenance: Intervention		In-house maintenance
Assumed equipment lifetime reduction:	39%	0%	0%	0%
	(1)	(2)	(3)	(4)
**Fixed Cost (in t = 0)**				
*Management, contracted*				
IT-System	–	181,196	181,196	181,196
Transport	–	71,199	71,199	71,199
Office Space	–	6875	6875	6875
*Investment, in-house*				
Workshops	–	–	–	421,830
Tools	–	–	–	118,754
*Preventive maintenance, contracted*				
Rapid inventory assessment	–	251,355	251,355	–
Mobilization phase	–	159,663	159,663	–
***Total fixed cost***	***0***	***670,288***	***670,288***	***799,855***
**Operating Cost (in t = 1,2,3)**				
*Management, contracted*^a^				
IT-System	–	71,094	71,094	71,094
Car	–	17,977	17,977	17,977
Annual salaries		178,720	178,720	178,720
*Replacement cost*	4,924,275	3,003,808	3,003,808	3,003,808
*Maintenance cost*				
Invoiced program cost, contracted				
Preventive maintenance	–	465,777	–	–
Spare parts	–	27,914	27,985	–
Calculated program cost, contracted				
Preventive maintenance	–	–	72,168	–
Corrective maintenance	–	–	14,286	–
Calculated program cost, in-house				
Annual salaries	–	–	–	288,856
Spare parts and tests	–	–	–	185,349
***Total operating cost***	***4,924,275***	***3,765,360***	***3,386,037***	***3,745,804***
**Downtime Cost**				
***Total downtime cost***^b^	***104,547***	***65,925***	***not applicable***	
**Total program cost (NPV)**	***5,028,823***	***4,501,574***	***4,056,325***	***4,545,658***

*Notes*: Each cell reports net present value of total cost in 2016 I$ PPP over three years, calculated for the sum of 19 district hospitals from the two pilot regions. ^a^As maintenance management responsibilities do not include the procurement of new equipment, we do not include it in the management cost estimates. ^b^As we have no reliable estimate for downtime cost under inhouse maintenance, it is only considered for the comparison of replacement with contracting out scenarios. We assume downtime cost to be equal between full in-house and contracted out maintenance

In the hypothetical no maintenance scenario, we assume broken devices to be exchanged for new devices when a fault is detected (column 1 in Table 2). This scenario well reflects the situation in Nepalese health facilities prior to the pilot. As such a scenario does not involve maintenance activities, its main cost driver is more frequent device replacement.

The hypothetical in-house maintenance scenario delegates maintenance activities to the hospitals, while keeping up an administrative unit for monitoring and steering at the federal ministry (column 4 in Table 2). Such a decentralized scheme describes a common comparison scenario. It rests on three important assumptions. First, we assume that fixed and operational central management costs are overall the same for contracted-out and in-house maintenance. In-house maintenance requires less central planning than contracted-out maintenance, but smaller performance incentives might increase the need for oversight. Second, we assume that both contracted-out and in-house maintenance perform equally well in keeping equipment downtime low. Third, we assume that there is sufficient biomedical technical expertise available in the country to employ at least one biomedical engineer per district hospital. For salaries as well as costs of spare parts, tests, and facilities, we rely on expert estimates from Nepal’s Physical Asset Management unit (Table A3 in the Appendix). To allow for a fair comparison, we recalculate operational maintenance cost of Nepal’s contracted-out maintenance scheme (see column 3 in Table 2). Instead of drawing on invoiced maintenance cost only, where training of in-house technicians makes up a considerable share, we use corrective maintenance entries from the MMIS and assume a reasonable flat rate for every single scheduled maintenance inspection.

## Results

### No versus contracted-out maintenance

Nepal’s contracted-out scheme requires initial investments of 670,288 I$ PPP for technical and management infrastructure. After three years, operational maintenance cost – for both preventive and corrective maintenance – amount to 493,762 I$ PPP (11.0% of total cost). Operational management and downtime cost account for 267,790 I$ PPP (5.9% of total cost) and 65,925 I$ PPP (1.5% of total cost), respectively. Together, these cost items add up to almost 1.5 million I$ PPP, which is still less than half of the replacement cost (3 million I$ [PPP]). In the absence of any maintenance (scenario A in Table 2), replacement cost amounts to 4,924,275 I$ PPP. In sum, this substantial cost difference in replacement cost leads to a cost advantage of contracted-out maintenance over a no maintenance approach by 527,249 I$ PPP, after three years. Please note, that this comparative result mainly rests on the 39% lifetime reduction rate we have assumed. Our robustness check in Fig. A1 in the Appendix shows that contracted-out maintenance outperforms no maintenance in a three-year time horizon as long as the lifetime reduction rate is above 30%.

### Contracted-out versus in-house maintenance

Fixed and operational cost of contracted-out maintenance are 129,566 I$ PPP (16.2%) and 359,767 I$ PPP (9.6%) less than for in-house maintenance after three years. In-house maintenance includes building a workshop and equipping it with tools, accumulating to about 799,855 I$ PPP. Maintenance cost includes technical in-house staff and requires hardware costing about 474,205 I$ PPP. Thus, the cost difference between contracted-out and in-house maintenance is mainly driven by the much higher cost of salaries and spare parts of in-house maintenance.

### Additional results

To put the total numbers in context, we break down cost to the hospital level. To this end we assume that all fixed and operational costs are equally shared by the number of district hospitals and accordingly divide total program costs by the number of 19 district hospitals. Again, we consider the net-present value over a three-years period. According to Table A4 in the Appendix, total per hospital cost for a no maintenance approach and contracted-out maintenance amount to about 264,675 I$ PPP and 236,925 I$ PPP, respectively. Assuming hospitals do not have to contribute to neither fixed nor operational management cost, per hospital cost are 264,675 I$ PPP and 209,185 I$ PPP, for no maintenance and contracted-out maintenance, respectively.

**Fig. 1:**
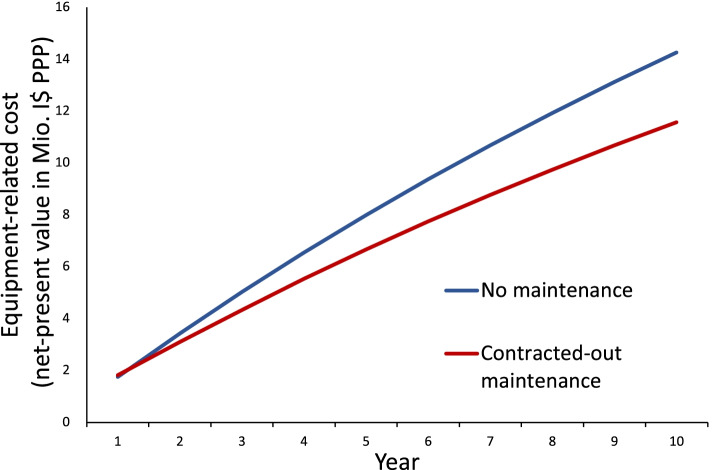
Comparative cost assessment for a ten-year time horizon. Notes: Abbreviations: mio. I$, Million International Dollars (Purchasing Power Parity)

Calculating program cost for a longer time horizon provides a second extension to our main analysis. Figure 1 illustrates how total equipment-related program cost for a no maintenance and contracted-out scheme evolve over a ten-years period. After the first year, no maintenance is 223,140 I$ PPP cheaper than contracted-out maintenance. For subsequent years, full replacement costs accumulate disproportionally fast and after 10 years exceed total contracted-out maintenance costs by 2,529,639 I$ PPP (17.7%). One key result of this extended analysis is that the additional investments of contracted-out maintenance already breaks-even after two years.

## Discussion

For our study setting in Nepal, contracted-out maintenance shows the highest cost efficiency across three analyzed medical equipment maintenances modes. Its cost advantage mainly stems from lower operating cost, thus, the cost advantage of contracted-out maintenance increases with program duration. Similarly, contracted-out maintenance’s relative cost advantage likely increases further when geographically upscaling the program.

In our study we emphasize the importance of incorporating downtime cost into the analysis when comparing scenarios with different outcomes [17]. The empirical literature on medical equipment management is scarce and there is no established benchmark for measuring the benefits from equipment downtime reduction. In a first attempt, we approximate the disutility of downtime by missed hospital revenue. We find contracted-out maintenance to be effective in reducing downtime cost when compared with no maintenance. Even if downtime cost from missed equipment-based revenues amount to less than 2% of total cost it may serve as an important benchmark outcome by itself: minimizing equipment downtime to improve quality of care and reduce treatment-induced health risks seems to be a worthwhile goal from a health systems planner’s perspective. In this regard Nepal provides a relevant context. In the absence of any maintenance scheme Nepali public hospitals were unable to fix even minor equipment malfunctions and suffered from a lack of equipment-related staff training [6]. As a large share of complex medical equipment in resource-constrained settings is donated through bilateral or multilateral development cooperation, our insights are relevant for both policy makers and funding institutions [18]. In-house maintenance is a common and thus policy relevant scenario. Our calculations provide a first cautious attempt to include such a scenario into a comparative analysis. Nevertheless, the cost items rely on hypothetical measures from Nepal’s Physical Asset Management unit and should be interpreted respectively.

This study is the first comprehensive costing study of medical equipment maintenance in a resource-constrained country. The quantitative literature on medical equipment in low- and lower-middle-income countries is very limited; most studies either provide simple equipment inventories or purely qualitative attempts to explore maintenance practices [5, 7–9]. However, many low-income countries lack a proper medical equipment maintenance policy and the capability to implement any of the literature’s recommendations [18, 19]. By taking a comprehensive approach and including no maintenance as a benchmark, this paper seeks to include this reality into the analysis. A favorable data situation enables us to address the high data requirements needed for such an exercise. Specifically, we include detailed information on device asset values and lifetimes, maintenance activities, equipment-related service quantities and prices for 19 district hospitals and six medical devices. This compares favorably to existing empirical studies of medical equipment maintenance, which consider not more than two hospitals or investigate only incremental changes to existing maintenance programs [20–23].

Our findings on the comparative advantage of contracted-out over in-house maintenance contradict similar studies within an university hospital setting in the United States and Brazil [21, 22]. Both these studies favor in-house maintenance over contracted-out maintenance. The reason for these different results might be that engineering expertise is much more available in university hospitals of high and upper-middle-income countries than in public hospitals of lower-middle-income countries.

It is important to note the following limitations of our study. First, we were not able to directly measure the difference in lifetime between well-maintained and non-maintained devices. As often the case in resource-constrained settings, Nepal has established an asset management inventory on medical equipment very recently and the pilot was too short to track changes in equipment lifetime [24]. To this end, we have to rely on estimates from the published literature [14]. Thus, the 39% lifetime reduction rate should be taken with caution. As our robustness check reveals, contracted-out maintenance’s relative cost advantage holds for an assumed equipment lifetime reduction greater than 30% (Fig. A1 in the Appendix). Second, we cannot directly observe how much time passed between the breakdown of a device and the malfunction being reported to the maintenance support unit. Hence, we consider the time passed between a corrective maintenance job request and the equipment’s last inspection and assume that 15% of such a time window is effective equipment downtime. Changing the time lag to 5% or 25% of the time passed since the last inspection does not alter our qualitative results (Table A5 in the Appendix). Third, it’s important to note that data quality from management information systems may vary substantially, depending on the context, including underlying data entering procedures, built-in data validations, incentives for misreporting and others. We conducted careful quality and plausibility checks with our data from Nepal’s Health Management Information System (HMIS) and Maintenance Management Information System (MMIS) and are confident that they do not draw a skewed picture of the reality. However, we want to emphasize the potential inherent limitations that arise by using management information system data.

As with many empirical studies, a final limitation is that some of our results might not be easily applicable to other circumstances. The pilot catchment area covers only about seven million people, roughly one quarter of Nepal’s total population. Due to its relatively low operational cost, we expect contracted-out maintenance to perform even better when being scaled up to the country level. This motivates the cost evaluation of Nepal’s country-wide contracted-out maintenance scheme, which has been implemented in 2016, for future research. It is worth noting that Nepal’s terrain, with many hard-to-reach areas, may constitute a case where maintenance cost is relatively high compared to other countries.

## Conclusion

To summarize, it is remarkable that contracted-out maintenance has by far the lowest cost, even when initial investments are considered, while reducing equipment downtime. Thus, this study confirms contracted-out maintenance as a cost-efficient tool to increase equipment availability in health facilities for resource-constrained settings. Future studies would benefit from using country-specific databases on equipment lifetime. In addition, further research may widen the scope by looking into the role of in-house staff training and the central Physical Assets Management Policy [25].

## Supplementary Information

**Additional file 1.** Data sources, assumptions, and robustness checks. Information on data sources (Tables A1, A6 & A7), assumptions (Table A2 & A3), robustness checks (Fig. A1 & Table A5), and additional results (Tables A4 & A8).

**Additional file 2.** Full cost calculations and formatted data. Detailed steps of the cost calculation and all data needed for replication in pre-processed format.

## Data Availability

The dataset supporting the conclusions of this article is included within the article (and its additional file(s)) with exception of unformatted maintenance job-cards. Job-card data is available from the corresponding author on reasonable request.

## Abbreviations

HMIS: Health Management Information System
MMIS: Maintenance management information system
I$ (PPP): International Dollar (Purchasing Power Parity)
