# Supplementary material for: Maintenance versus replacement of medical equipment: a cost-minimization analysis among district hospitals in Nepal
Source: BMC Health Serv Res. 2022 Aug 12;22:1023. doi: 10.1186/s12913-022-08392-6 (PMC9373529; doi:10.1186/s12913-022-08392-6)
Supplement: Supplementary file 1 — Additional file 1. Data sources, assumptions, and robustness checks. Information on data sources (Tables A1, A6 & A7), assumptions (Table A2 & A3), robustness checks (Fig. A1 & Table A5), and additional results (Tables A4 & A8). [file 12913_2022_8392_MOESM1_ESM.docx]

Additional file 1. Data sources, robustness, further results

Appendix to: To make, buy, or do nothing: A costing study of medical equipment maintenance schemes in Nepal

Michael Hillebrecht^1,2,3^, Constantin Schmidt^1^, Bhim Prasad Saptoka^4,5^, Josef Riha^6^, Matthias Nachtnebel^7^, Till Bärnighausen^1,8,9,10^

^1^ Heidelberg Institute of Global Health (HIGH), Medical Faculty and University Hospital, Heidelberg University, Heidelberg, Germany.

^2^ South-Asia Institute, Heidelberg University, Germany.

^3^ Deutsche Gesellschaft für Internationale Zusammenarbeit (GIZ) GmbH, Eschborn, Germany.

^4^ Health Coordination Division, Ministry of Health and Population, Kathmandu, Nepal.

^5^ Division of Infectious Diseases and Tropical Medicine, Center for International Health, University Hospital Munich, Ludwig-Maximilians-University, Munich, Germany.

^6^ management4health, Frankfurt, Germany

^7^ Health and Social Protection Asia, KfW Development Bank, Frankfurt am Main, Germany.

^8^ Africa Health Research Institute (AHRI), Somkhele, KwaZulu-Natal, South Africa.

^9^ MRC/Wits Rural Public Health and Health Transitions Research Unit (Agincourt), School of Public Health, Faculty of Health Sciences, University of the Witwatersrand, Johannesburg, South Africa.

^10^ Center for Population and Development Studies, Harvard University, Cambridge, MA, USA.

Corresponding Author:

Michael Hillebrecht (*Michael.hillebrecht@giz.de*)

**List of Figures**

[***Figure A1: Comparison of net present values for different lifetime reductions*** 5](#_Toc75179747)

**List of Tables**

[***Table A1: Cost components*** *3*](#_Toc75179750)

[***Table A2: List of assumptions for the management cost split*** *4*](#_Toc75179751)

[***Table A3: Physical Asset Management cost estimates*** *7*](#_Toc75179752)

[***Table A4: Per Hospital cost of no-maintenance and contracted-out maintenance*** *7*](#_Toc75179753)

[***Table A5: Cost of different downtime scenarios*** *7*](#_Toc75179754)

[***Table A6: List of devices for maintenance management pilot*** *8*](#_Toc75179755)

[***Table A7: Public Health Facilities in the Two Pilot Regions*** *8*](#_Toc75179756)

[***Table A8: Cost of Different Maintenance Scenarios in Rupee*** *9*](#_Toc75179757)

***Table A1: Cost components***

| **Cost Type** | | |  |  |  | | **Explanation** |  | **Data Source** |
| --- | --- | --- | --- | --- | --- | --- | --- | --- | --- |
|  |  |  |  |  |  | |  |  |  |
| **Fixed Cost** | | |  |  |  | |  |  |  |
|  | *Management, contracted* | | | | | Cost of setting up the central administrative unit ("Physical Assets Management"), who hires and monitors maintenance contractors: IT-system, transport, and office space. | |  | MMIS |
|  |  |  |  |  |  | |  |  |  |
|  | *Investment, in-house* | | | |  | | Cost of setting up a workshop in each health facility and equipping it with tools |  | MMIS |
|  |  |  |  |  |  | |  |  |  |
|  | *Maintenance, contracted* | | | | | Cost of launching program (mobilization phase): recruitment of personnel, workshop setup, purchase of vehicles, tools, initial spares stock, calibration equipment, stakeholder meetings; and device inventory (Rapid Inventory Assessment). | |  | MMIS, m4H, BoQ, Consultant (for prices) |
|  |  |  |  |  |  | |  |  |  |
| **Operating Cost** | | | |  |  | |  |  |  |
|  |  |  |  |  |  | |  |  |  |
| Overhead Cost | | | |  |  | |  |  |  |
|  | *Management, contracted* | | | | | Cost of setting up the central administrative unit ("Physical Assets Management"): IT-system, transport, and salaries | |  | MMIS |
|  |  |  |  |  |  | |  |  |  |
| Direct Cost | | |  |  |  | |  |  |  |
|  | *Replacement cost* | | |  |  | | Cost of depreciation of the medical devices |  | BoQ, Consultant (for prices), Halbwachs2000 |
|  |  |  |  |  |  | |  |  |  |
|  | *Maintenance cost* | | |  |  | | Cost of maintenance activities |  | m4H, BoQ, Consultant (for prices), PLAMHAS |
|  |  |  |  |  |  | | *Contracting out*: preventive maintenance inspections and spare parts |  |  |
|  |  |  |  |  |  | | *In-house maintenance*: salaries, spare parts, and tests |  |  |
|  |  |  |  |  |  | |  |  |  |
|  | *Downtime cost* | | |  |  | | Foregone income due to non-operational devices |  | Social Health Security Program, HMIS, MMIS |
|  |  |  |  |  |  | |  |  |  |

Notes: BoQ; Bill of Quantity; HMIS; Health Management Information System (General data base on public hospitals in Nepal); m4H; Management for Health; MMIS, Maintenance Management Information System (Internal data platform of maintenance management pilot).

**Table A2: List of assumptions for the management cost split**

|  |  |  | **Share** |
| --- | --- | --- | --- |
| ***Start-up cost*** | | |  |
|  | IT-System | | 0.7 |
|  | Car |  | 0.5 |
|  | Rooms | | 1 |
|  | Rapid Inventory Assessment | | 1 |
|  | Mobilization Phase | | 0.3 |
| ***Running cost*** | | |  |
|  | Staff | |  |
|  |  | RTM | 1 |
|  |  | PAM Engineer | 1 |
|  |  | PAM Chief | 0.5 |
|  | IT-System | | 0.3 |
|  | Car |  | 0.5 |

Notes: Share represents the share of total cost observed during the maintenance pilot we estimate would arise if only the devise we consider were part of the pilot. E.g.: 70% of the cost for IT-Systems for the whole pilot are considered in the cost calculation for the maintenance of devices we consider.

***Figure A1: Comparison of net present values for different lifetime reductions***

*Notes: Abbreviations: I$ PPP, International Dollar Purchasing Power Parity; NPV, Net Present Value.*

**Table A3: Cost estimates for in-house maintenance**

|  | **Resources needed** |
| --- | --- |
| Total maintenance staff salary per Year | 18,751 |
| Tools (I$ [PPP]) | 6,250 |
| Spares (I$ [PPP]) | 10,938 |
| Calibration and Testing tools (I$ [PPP]) | 6,250 |
| Space (sq m) | 12 |
| Space (sq ft) | 129 |
| Construction cost (5500 per sq ft, I$ [PPP]) | 22,202 |
| Rooms | 1 |

Notes: Estimates are for a 15-bed hospital and were provided by the Physical Asset Management unit of Nepal’s Ministry of Health.

**Table A4: Per Hospital cost of no-maintenance and contracted-out maintenance**

|  |  | **Fixed Cost** | **Operating Cost** | **Downtime Cost** | **Total Program Cost** | **Cost without management** |
| --- | --- | --- | --- | --- | --- | --- |
|  |  |  |  |  |  |  |
| **No maintenance scenario (A)** |  | 0 | 259,172 | 5,502 | 264,675 | 264,675 |
| **Contracted-out maintenance pilot intervention (B)** |  | 35,278 | 198,177 | 3,470 | 236,925 | 209,185 |
|  |  |  |  |  |  |  |

Notes: All cost reported are NPVs over 3 years. Maintenance scenarios as described in methods section.

**Table A5: Cost of different downtime scenarios**

| **Downtime Cost (NPV over 3 Years)** | **Assumed time-lag** | | |
| --- | --- | --- | --- |
|  | **5%** | **15%** | **25%** |
| **No-maintenance scenario (A)** | 34,849 | 104,547 | 174,246 |
| **Maintenance contracted-out pilot intervention (B)** | 21,975 | 65,925 | 109,875 |
| Notes: Time-lag is calculated as fraction of time passed between last corrective maintenance visit and prior maintenance inspection, as described in methods section. Values in International Dollars Purchasing Power Parity.. | | | |
|  |  |  |  |

**Table A6: List of devices for maintenance management pilot**

|  | **Equipment Category** | | **Equipment Types Included** | | | **Total Number of Devices** |  |  |
| --- | --- | --- | --- | --- | --- | --- | --- | --- |
|  |  |  |  |  |  |  |  |  |
|  |  |  |  |  |  |  |  |  |
| **1** | **Laboratory equipment** | | analyzer, centrifuge, microscope | |  | 776 |  |  |
|  |  |  |  |  |  |  |  |  |
| **2** | **Cold chain equipment** | | refrigerator, freezer | |  | 921 |  |  |
| **3** | **Surgical equipment** | | anesthesia unit, electrosurgical unit, operating light, oxygen concentrator, sterilizing unit, operating table | |  | 1,000 |  |  |
|  |  |  |  |  |  |  |  |  |
|  |  |  |  |  |  |  |  |  |
|  |  |  |  |  |  |  |  |  |
|  |  |  |  |  |  |  |  |  |
| **4** | **Monitoring** |  | ecg, monitor |  |  | 1,075 |  |  |
| **5** | **Ultrasound imaging** | |  |  |  | 1,108 |  |  |
| **6** | **Xray imaging** |  |  |  |  | 1,132 |  |  |
| *Notes: The total number of devices includes all devices in each equipment category for all hospitals participating in the pilot. Abbreviations: ecg, electrocardiogram.* | | | | | | | |  |
|  |  |  |  |  |  |  |  |  |

**Table A7: Public Health Facilities in the Two Pilot Regions**

| **Seti** |  | |  | **Bheri** | | |  | |
| --- | --- | --- | --- | --- | --- | --- | --- | --- |
| 1 | Kailali | | Seti Zonal Hospital | 1 | Surkhet | Mid Western Regional Hospital Surkhet | | |
| 2 | Achham | | Achham District Hospital | 2 | Banke | Bheri Zonal Hospital | | |
| 3 | Bajhang | | Bajhang District Hospital | 3 | Dailekh | Dailekh District Hospital | | |
| 4 | Bajura | | Bajura District Hospital | 4 | Bardia | Gulariya Hospital Bardiya | | |
| 5 | Doti | | Doti District Hospital | 5 | Jajarkot | Jajarkot District Hospital | | |
| 6 | Kailali | | Tikapur District Hospital |  |  |  | | |
| **Mahakali** | | |  | **Karnali** | |  | | |
| 1 | | Kanchanpur | Mahakali Zonal Hospital | 1 | Jumla | Kharnali Zonal Hospital | | |
| 2 | | Baitadi | Baitadi District Hospital | 2 | Dolpa | Dolpa District Hospital | | |
| 3 | | Dadeldhura | Dadeldhura District Hospital | 3 | Humla | Humla District Hospital | | |
| 4 | | Darchula | Darchula District Hospital | 4 | Kalikot | Kalikot District Hospital | | |
|  | |  |  | 5 | Mugu | Mugu District Hospital | | |
| **Rapti** | |  |  |  |  |  | | |
| 1 | | Dang | Rapti Sub-Regional Hospital |  |  |  | | |
| 2 | | Dang | Rapti Zonal Hospital |  |  |  | | |
| 3 | | Pyuthan | Pyuthan District Hospital |  |  |  | | |
| 4 | | Rolpa | Rolpa District Hospital |  |  |  | | |
| 5 | | Rukum | Rukum District Hospital |  |  |  | | |
| 6 | | Salyan | Salyan District Hospital |  |  |  | | |
| *Notes: Shows all hospitals included into the maintenance program. Hospitals on white background were included into the analysis.* | | | | | | | |  |

**Table A8: Cost of Different Maintenance Scenarios in Rupee**

| **Cost scenario:** | | |  |  |  |  | **A** | **B** | | **C** |
| --- | --- | --- | --- | --- | --- | --- | --- | --- | --- | --- |
|  |  |  |  |  |  |  | **No maintenance** | **Maintenance contracted-** | | **In-house** |
|  |  |  |  |  |  |  |  | **out: pilot intervention** | | **maintenance** |
| **Assumed equipment lifetime reduction:** | | | | | |  | **39%** | **0%** | **0%** | **0%** |
|  |  |  |  |  |  |  | **(1)** | **(2)** | **(3)** | **(4)** |
|  |  |  |  |  |  |  |  |  |  |  |
| **Fixed Cost (in t=0)** | | | |  |  |  |  |  |  |  |
|  | *Management, contracted* | | | |  |  |  |  |  |  |
|  |  | IT-System | |  |  |  | - | 5,798,063 | 5,798,063 | 5,798,063 |
|  |  | Transport | |  |  |  | - | 2,278,282 | 2,278,282 | 2,278,282 |
|  |  | Office Space | |  |  |  | - | 220,000 | 220,000 | 220,000 |
|  | *Investment, in-house* | | | |  |  |  |  |  |  |
|  |  | Workshops | |  |  |  | - | - | - | 13,498,056 |
|  |  | Tools | |  |  |  | - | - | - | 3,800,000 |
|  | *Preventive maintenance, contracted* | | | | |  |  |  |  |  |
|  |  | Rapid inventory assessment | | | |  | - | 8,043,070 | 8,043,070 | - |
|  |  | Mobilization phase | | |  |  | - | 5,109,017 | 5,109,017 | - |
|  | ***Total fixed cost*** | | |  |  |  | ***0*** | ***21,448,432*** | ***21,448,432*** | ***25,594,401*** |
|  |  |  |  |  |  |  |  |  |  |  |
| **Operating Cost (in t=1,2,3)** | | | | |  |  |  |  |  |  |
|  | *Management, contracted** | | | |  |  |  |  |  |  |
|  |  | IT-System | |  |  |  | - | 2,274,920 | 2,274,920 | 2,274,920 |
|  |  | Car |  |  |  |  | - | 575,236 | 575,236 | 575,236 |
|  |  | Annual salaries | |  |  |  |  | 5,718,821 | 5,718,821 | 5,718,821 |
|  | *Replacement cost* | | |  |  |  | 157,570,952 | 96,118,280 | 96,118,280 | 96,118,280 |
|  | *Maintenance cost* | | |  |  |  |  |  |  |  |
|  |  | Invoiced program cost, contracted | | | |  |  |  |  |  |
|  |  |  | Preventive maintenance | | |  | - | 14,904,311 | - | - |
|  |  |  | Spare parts | |  |  | - | 895,476 | 895,476 | - |
|  |  | Calculated program cost, contracted | | | | |  |  |  |  |
|  |  |  | Preventive maintenance | | |  | - | - | 2,309,278 | - |
|  |  |  | Corrective maintenance | | |  | - | - | 457,138 | - |
|  |  | Calculated program cost, in-house | | | |  |  |  |  |  |
|  |  |  | Annual salaries | |  |  | - | - | - | 9,243,046 |
|  |  |  | Spare parts and tests | |  |  | - | - | - | 5,930,954 |
|  | ***Total operating cost*** | | |  |  |  | ***157,570,952*** | ***120,487,045*** | ***108,349,150*** | ***119,861,257*** |
|  |  |  |  |  |  |  |  |  |  |  |
| **Downtime Cost** | | | |  |  |  |  |  |  |  |
|  | ***Total downtime cost***** | | | |  |  | ***3,345,395*** | ***2,109,525*** | ***not applicable*** | |
|  |  |  |  |  |  |  |  |  |  |  |
| **Total program cost (NPV)** | | | |  |  |  | ***160,916,346*** | ***144,045,002*** | ***129,797,582*** | ***145,455,658*** |
|  |  |  |  |  |  |  |  |  |  |  |
| *Notes: Each cell reports net present value of total cost in 2016 NPR over three years, calculated for the sum of 18 district hospitals from the two pilot regions. *As maintenance management responsibilities do not include the procurement of new equipment, we do not include it in the management cost estimates. **As we have no reliable estimate for downtime cost under inhouse maintenance, it is only considered for the comparison of replacement and contracting out scenarios. Thus, we assume downtime cost to be equal between full in-house and contracted out maintenance.* | | | | | | | | | | |
